# Statistical methods for retrospective harmonization of longitudinal epidemiological data: a scoping review

**DOI:** 10.1007/s10654-026-01404-3

**Published:** 2026-05-28

**Authors:** Jiumeng Zhang, Jordan Behrendt, Tanja Schultz, Krasimira Aleksandrova, Khalid Iqbal, Iris Pigeot, Claudia Börnhorst

**Affiliations:** 1 Department of Statistical Methods in Epidemiology, Leibniz Institute for Prevention Research and Epidemiology – BIPS, Achterstraße 30, 28359 Bremen, Germany; 2 Faculty of Mathematics and Computer Science, University of Bremen, Bremen, Germany; 3 Department Epidemiological Methods and Etiological Research, Leibniz Institute for Prevention Research and Epidemiology – BIPS, Bremen, Germany; 4 Faculty of Human and Health Sciences, University of Bremen, Bremen, Germany

**Keywords:** Data harmonization, Data pooling, Integrative data analysis, Retrospective harmonization, Longitudinal data

## Abstract

Data harmonization is a prerequisite for joint cohort analyses. In this review, we aim to identify and contrast statistical methods for retrospective harmonization of longitudinal data. We performed a scoping review following the Preferred Reporting Items for Systematic Reviews and Meta-Analyses extension for Scoping Reviews guidelines. Studies were included if they described statistical methods for retrospectively harmonizing longitudinal data at the participant level. From 35 included papers out of 1,234 hits, we identified three types of statistical methods applicable to tabular data commonly collected in longitudinal epidemiological studies (e.g., questionnaires): (1) distribution-based methods, (2) the proportion score model, and (3) latent variable models. Our results suggest that the suitability of a statistical harmonization method mainly depends on the measurement scales of the original variables as well as on the type of target variable (directly measurable vs. latent). The chosen harmonization method influences how missing subsets of variables are addressed. None of the included studies applied more automated approaches such as machine learning-based procedures for deriving a harmonized dataset. Based on our findings, we present a roadmap that can guide researchers in selecting the most appropriate statistical method for a specific harmonization task and in handling variables collected only in a subset of studies. Data harmonization is still a demanding task that requires the development and application of novel tools for automating the procedures.

**Supplementary Information:**

The online version contains supplementary material available at https://doi.org/10.1007/s10654-026-01404-3.

Box 1: Glossary Data harmonization: a process to achieve (or improve) comparability of putatively equivalent measures collected by different studies on different individuals

Retrospective harmonization: approaches that process study-specific variables into a common format applied to existing datasets

Integrative data analysis (IDA): a form of data pooling which involves the simultaneous analysis of data pooled from two or more independently conceived datasets

Simple algorithmic transformation: applying rule-based algorithms to recode study-specific variables (e.g. recoding of categories)

Simple calibration: rescaling continuous variables with a known calibration model (e.g. convert weight in kilograms to pounds)

Concept block: search terms that are organized into the same thematic group that will be combined with other search terms when building a search strategy in databases

Pooled dataset: a single dataset created by combining conceptually similar and comparable individual-level data from multiple sources

## Introduction

Over recent decades, large-scale cohort studies have been initiated to collect longitudinal health data for understanding the association between risk factors and diseases, which provide a great research resource [1–4]. The growing demand for data sharing in response to increased research collaboration has driven the establishment of frameworks like the FAIR (Findable, Accessible, Interoperable, and Reusable) data principles, published in 2016 [5]. These developments provide more opportunities for the joint analyses of cohort data. Joint analyses offer many advantages including efficiency (i.e., reuse of extant longitudinal data from cohort studies which are often expensive and labor-intensive), increased power (i.e., larger sample size), and the potential to address novel questions that cannot be answered by a single contributing study (e.g., extended observation periods) [6]. However, data collection instruments are usually developed targeting the individual study scope and current research needs, which leads to incompatibility when trying to combine existing datasets. Therefore, statistical procedures and algorithms are required to achieve (or improve) comparability of the putatively equivalent measures collected by different studies on different individuals, i.e. to harmonize the data [7].

Generic guidelines (so-called Maelstrom Guidelines) have been developed outlining the procedural steps for retrospective data harmonization [8]. In addition, several efforts such as the (German) National Research Data Infrastructure (e.g. NFDI4Health for Personal Health Data) and the Melbourne Children’s LifeCourse initiative are launched to foster the FAIRification of epidemiological data, where harmonization is one important part [9, 10]. However, little guidance exists on how to select suitable methods and algorithms to process study-specific variables into a common format. Typically, simple methods (i.e., simple algorithmic transformation and simple calibration) are commonly applied for data harmonization because of their ease of use and quick implementation [8, 11–21]. When applying these simple methods, the transformation rules are often developed manually by comparing the content and scale of each pair of variables based on the judgments of the investigators. Since this process could be quite time consuming and labor intensive, automated procedures would be more efficient when harmonizing a large number of variables across studies. Furthermore, sophisticated statistical methods or algorithms based on machine learning may be useful to address complex issues (e.g., when harmonizing latent constructs such as well-being or cognition) and to minimize information loss.

Harmonizing longitudinal epidemiological data presents an additional challenge because assessment instruments may change over time (e.g., due to technological advances). Consequently, data must be harmonized not only across studies but also over time. Another challenge that arises in real world harmonization practices is how to handle missingness in the pooled dataset that results from variables collected only in a subset of studies.

Despite the acknowledged importance and challenges of data harmonization in epidemiological research, there is a notable gap in the literature concerning the discussion and comparison of (1) statistical methods and machine learning-based algorithms for harmonizing variables across studies as well as over time and (2) methods to handle systematically missing data in the pooled dataset. Previous reviews on methods for data harmonization have been conducted in some domains such as radiomics, gene expression and pathology [22–24]. These reviews mainly explored approaches that are highly targeted towards the problems associated with specific research data (such as inconsistencies of magnetic resonance imaging datasets from different protocols and/or scanners, technical batch effects in genotyping data) during the integration of datasets. Therefore, it is challenging to generalize these approaches to datasets collected through different assessment tools (e.g., questionnaires).

In the present scoping review, we aimed to identify and contrast statistical methods for retrospective harmonization of longitudinal epidemiological data and to provide guidance on how to choose the method suited best for a specific harmonization task while minimizing the loss of information during the harmonization process. The results were summarized in a roadmap that may guide researchers aiming to retrospectively harmonize data across multiple cohorts and over time to select the most suitable statistical method.

## Methods

### Search strategy and eligibility criteria

The literature search was conducted using three search engines: PubMed, Web of Science and IEEE Explore to identify statistical procedures as well as algorithms based on machine learning. All databases were searched from January 1, 2000, to December 25, 2023. Articles were restricted to those available in English language. The search results from the databases were supplemented by papers identified from the reference lists of included papers.

In addition to methodological articles describing statistical methods for data harmonization, we also checked the reported statistical methods for data harmonization in large pooling studies. We included articles that meet the following two inclusion criteria:

1. propose, summarize or apply statistical methods for data harmonization when pooling individual-level observational data from two or more independently conceived datasets,
2. are based on longitudinal epidemiological data.

**Fig. 1:**
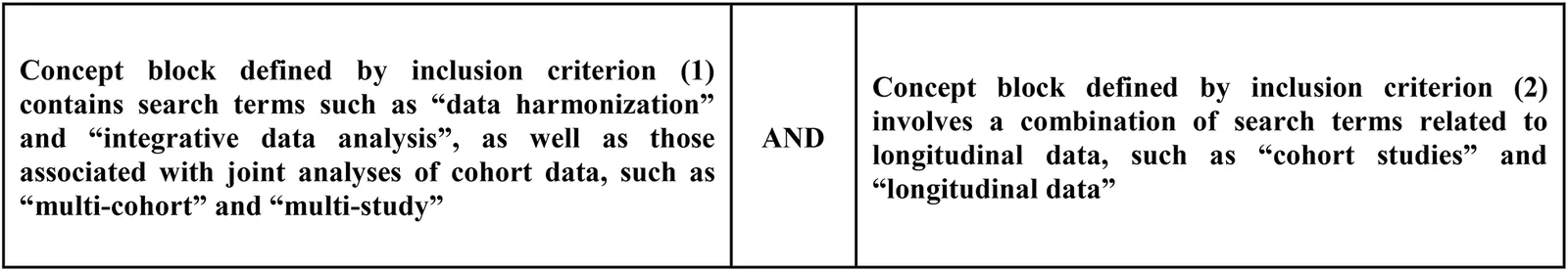
Schematic display of the search strategy

There were no standard keywords or subject headings used in bibliographic databases to identify literature describing statistical processing methods for data harmonization. The search strategy was generated based on the above inclusion criteria. Search terms were collected for each inclusion criteria in a concept block. Within each concept block, search terms were stringed together with Boolean operator “OR”. The search queries were constructed by combining these search terms in the two concept blocks using Boolean operator “AND” (See Fig. 1 for illustration). Studies were included if they described a methodology for retrospectively harmonizing longitudinal data at the individual level where we focused on more sophisticated statistical methods as well as automated procedures/tools for data harmonization. Studies using only simple methods such as recoding or simple calibration were not included as these are already well known and widely applied. Studies on methods that are highly targeted towards the integration of specific datasets such as magnetic resonance imaging (MRI) datasets or genotyping data were excluded. Previous reviews have already provided a comprehensive summary of computational and statistical methods for these scenarios [22–24], and these approaches cannot be easily adapted to epidemiological datasets collected based on other assessment instruments such as questionnaires. See Online Resource Appendix A and Appendix B for complete search strings and detailed exclusion criteria.

### Study selection and data extraction

Following the Preferred Reporting Items for Systematic Reviews and Meta-Analyses extension for Scoping Reviews (PRISMA-ScR) guideline [25], duplicate records in the search results were removed before title and abstract screening. Two reviewers conducted title and abstract screening independently based on the eligibility criteria and subsequently conducted the full-text screening. Articles remaining after the full-text review were selected for inclusion in the scoping review. In addition to the search based on the databases, papers identified from the reference list of included papers were also evaluated for eligibility using the same criteria.

For methodological articles summarizing statistical procedures for data harmonization without empirical example, information about the method used for data harmonization, availability of automated tools, as well as the evaluation metrics used to check the quality of the harmonized dataset was collected, when available. For large pooling studies and methodological papers with empirical examples for illustration, in addition to the above-mentioned information, the purpose for data pooling, total pooled sample size, age range in harmonized dataset, harmonized variable(s) and methods for dealing with missing values were extracted.

## Results

**Fig. 2:**
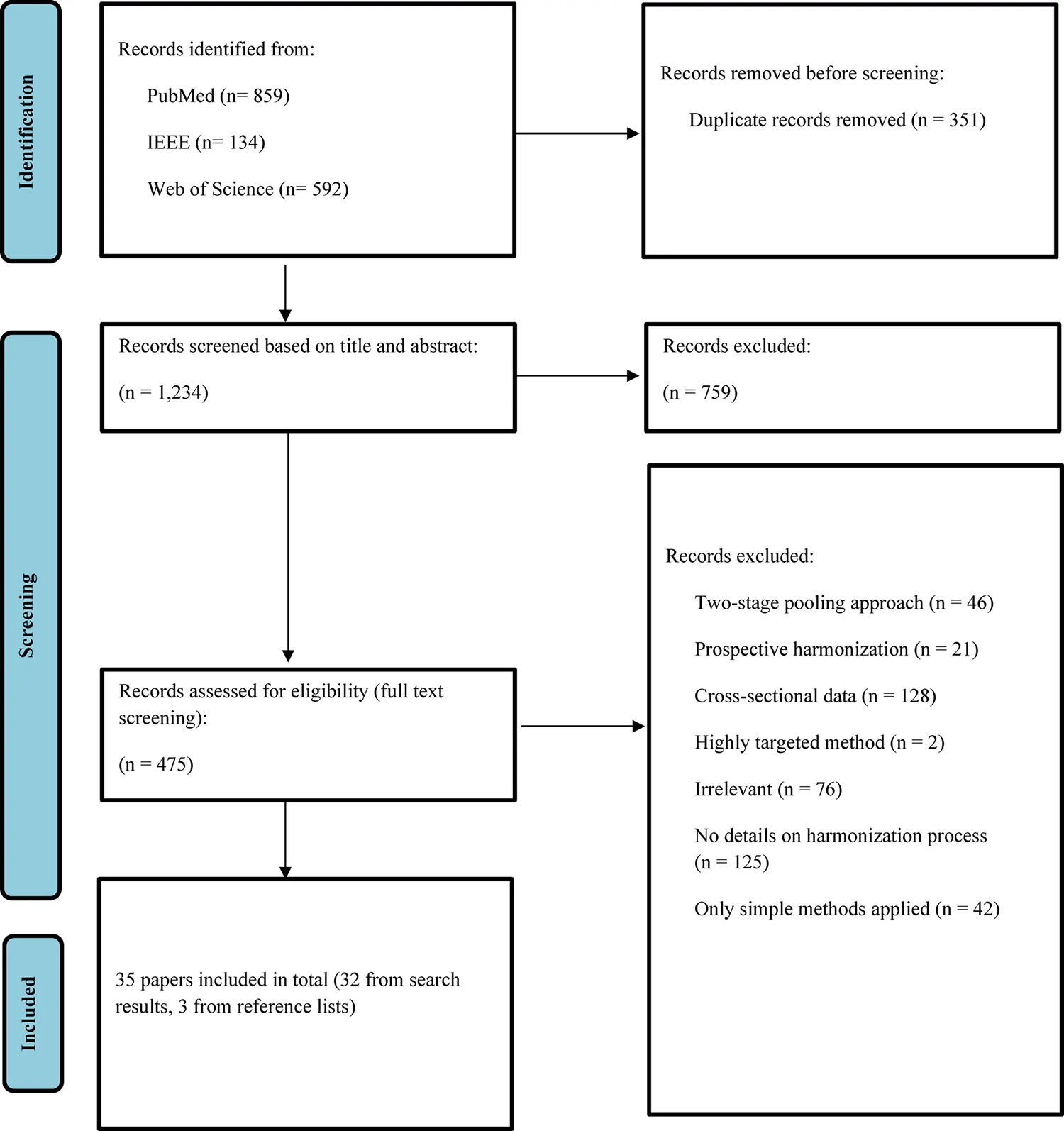
Flowchart depicting the selection process

As illustrated in the flowchart in Fig. 2, a total of 1,585 records were identified from the three databases. After removing of duplicates, 1,234 records remained for title and abstract screening. Applying the eligibility criteria, 475 records remained for full-text screening and finally 35 papers were included in the review (32 records from the search results and 3 articles identified from the reference lists of the included records).

Descriptive characteristics are provided in Online Resource Appendix C and Appendix D separately for 10 methodological papers summarizing statistical harmonization methods as well as for 25 pooling studies and methodological papers describing/proposing statistical harmonization methods with an empirical example for illustration. The methodological studies primarily focus on latent variable models related to topics such as psychometrics, where the main emphasis is on the study of latent constructs. The included pooling studies combine 2 up to 21 studies mainly aimed at increasing statistical power, extending the age range for developmental studies and enhancing the generalizability of findings.

Overall, three types of statistical methods were identified: (1) distribution-based methods (linear equating and equipercentile equating) [7, 8, 26–40], (2) the proportion score method [29, 41] and (3) latent variable models (linear factor analysis, item response theory, moderated non-linear factor analysis) [6–8, 26, 40–56] as detailed in the following. Table 1 provides a summary of identified statistical methods for data harmonization; a detailed description and application examples of the identified methods are given in Online Resource Appendix E.

**Table 1:** Summary of identified statistical harmonization methods

Harmonization scenario	Type	Harmonization method	Data adaptability	Advantages	Disadvantages
Target variable to generate is either observed variable or latent construct; only single-item measures available	Distribution-based methods	Linear equating [7, 8, 26–28, 30–40]	Continuous variables	• Only requires first two moments, easy to compute	• Requires the variable to be normally distributed
		Equipercentile equating [7, 8, 29, 31, 39]		• Could be used when variable has highly skewed or bimodal distribution	• Require large sample size
Target variable to generate is latent construct measured by multiple items; the harmonization team has access to item level data from contributing studies	Proportion score	Proportion score [26, 29, 41]	Binary variables	• Easy to calculate and interpret	• All endorsed items are weighted equally, and there is no allowance for differences across items within a scale • All items are assumed to equivalently define the construct both over time and across groups
Target variable to generate is latent construct measured by multiple items; the harmonization team has access to item level data from contributing studies	Latent variable model	Linear factor analysis (LFA) [7, 8, 40, 51, 52, 56]	Continuous indicator variables(items)	• Well known methodology with many software implementations • Relatively easy to implement compared with MNLFA	• Only allow the parameters of the model to be moderated by observed discrete variables • Requires at least one common item across datasets
		Item response theory (IRT) model [7, 8, 26, 40–48, 52, 55]	Binary/ordinal indicator variables(items)		• Only allow the parameters of the model to be moderated by observed discrete variables • Requires at least one common item across datasets • Requires large sample size
		Moderated non-linear factor analysis (MNLFA) [6–8, 40, 42, 49–54]	Indicator variables (items) of mixed type (e.g., binary, continuous, ordinal)	• Accommodates mixed item types • Allows the parameters of the model to be moderated by observed discrete variables and continuous variables	• Requires at least one common item across datasets • Requires large sample size • Computationally demanding and requires expertise in advanced statistical modeling

### Statistical methods for harmonizing longitudinal data

#### Distribution-based methods

Distribution-based methods rely on statistical transformations that align the distributions of variables across studies and over time. A well-known example of a distribution-based method is standardization. A detailed demonstration of how to apply these methods is available in [39]. These methods are typically applied in pooling studies involving single-item measures [27–38]. Single-item measures include not only measurements of directly observable variables (e.g., height or weight), but also single indicators or composite scores of latent constructs, such as the single-question instruments of happiness and life satisfaction in the well-being module from European Social Survey [57]. Distribution-based methods implicitly assume that variables from different studies are measuring the same target of interest and can be directly compared by aligning the distribution of the variables, such as mean, variance, or percentiles. Both, linear equating and equipercentile equating, are distribution-based harmonization methods:

Linear equating is a simple method for harmonizing variables by converting values of one variable to have the same mean and standard deviation as the other variable. It is easy to compute since it only requires the first two moments. Standardization based on z-scores and t-scores could be seen as a special case of linear equating. Despite its simplicity, linear equating assumes the variables to be normally distributed, which limits its applicability in some application scenarios.

Compared to linear equating, equipercentile equating aligns the distribution of variables based on their percentile ranks within the respective distributions, and it allows for non-linear transformations. Instead of aligning means and standard deviations, equipercentile equating matches the percentiles of one distribution with those of another. Unlike linear equating, equipercentile equating does not require variables to be normally distributed and could be used even if distributions of variables differ significantly in shape. However, equipercentile equating requires large sample sizes with sufficient variability to produce stable estimates of relative position. Therefore, it is more computationally intensive than linear equating and can be problematic in small datasets where accuracy of the percentile estimates is low.

#### Proportion score

The proportion score is a method for harmonizing latent constructs measured by multi-item scales. Examples of scales commonly used in epidemiological research include the Patient Health Questionnaire-9 (PHQ-9) for assessing the severity of depression [58] and the Mini-Mental State Examination (MMSE) for assessing cognitive performance [59]. Within a multi-item scale, $\:{\mathrm{y}}_{\mathrm{i}\mathrm{j}\mathrm{t}}\:$ is the response of the binary item *j* from individual *i* at time point *t*. When the original items are continuous or categorical, it could be converted to the binary format as follows (positively endorsed item indicates positive association with the underlying latent construct being measured):

$$\:{\mathrm{y}}_{\mathrm{i}\mathrm{j}\mathrm{t}}=\left\{\begin{array}{c}0\:\:not\:endorsed\:\:\:\:\:\:\:\:\:\:\:\:\:\:\:\:\:\:\:\\\:1\:positively\:endorsed\:\:\:\:\:\:\end{array}\right.$$

Then the latent construct could be harmonized at the factor level by creating person- and age-specific scale scores based on a set of binary items. The scores are calculated by adding up the number of positively endorsed items and dividing by the total number of available items for each person within each time point.

Although the proportion score is straightforward and directly interpretable, there are several disadvantages: First, all endorsed items are weighted equally, for instance, one item about “feeling sad” and one about “suicidal ideation” would contribute equally to an overall mental health score. Second, all items are assumed to define the construct in the same way across groups (such as age and study membership). This assumption is not realistic in some situations, e.g., the item assessing ‘cries easily’ would be treated as an equally valid indicator of depression for both children and adults. Despite these limitations, the proportion score is used to provide a quick and easy way to summarize test scores [26, 41].

#### Latent variable models

Latent variable models have been developed to measure unobserved latent constructs using observed indicators. Latent variable models are widely used in harmonizing latent construct variables [6, 41, 43–50, 52, 56]. Unlike distribution-based methods which harmonize at the level of single-item measures, both latent variable models and the proportion score method harmonize data at the factor level (i.e., use multiple observed indicator variables to create comparable factor scores across studies and over time). Since latent variable models require at least one common observed variable (i.e., item) across datasets, most pooling studies usually start with harmonization at the single item level (i.e., using simple algorithmic transformation to make the original item scales comparable). Key advantages of latent variable models include the following: First, for identical or easily harmonized items, it is possible to test whether participants from different studies (and/or over time within studies) respond to the items in the same way (so-called differential item functioning test). Second, for items from individual studies that cannot be directly harmonized across studies, we can often retain these unique items when deriving a score for the latent construct to improve measurement within a given study by retaining and incorporating more information when fitting the measurement model [6]. Therefore, these models are particularly useful for harmonizing longitudinal data across studies when different assessment instruments are used across studies and/or over time to measure the same latent construct.

In linear factor analysis (LFA), observed indicator variables (i.e., items) are modeled as linear functions of one or more latent factors. LFA can evaluate whether the measure reflects the same construct at each assessment (i.e., evaluate whether measurement invariance holds) and based on measurement invariance derive comparable latent factor scores across the studies. A detailed description of evaluating factorial invariance in longitudinal studies could be found in [60]. However, compared with other latent variable modeling methods, LFA is not widely used in data harmonization since it assumes linear relationships between the observed variables and the latent factors, which is quite restrictive in many situations.

Item response theory (IRT) refers to a broad set of non-linear statistical models that estimates the probability of a given response based on the respondent’s level of a latent trait (e.g., ability, mental health) and the psychometric properties of the item (e.g., items’ ability to discriminate among individuals’ levels of latent trait) [41]. The two parameter logistic IRT model (2PL) is one of the most widely used forms of the latent variable model in data harmonization for dichotomously scored items [41–46]. However, the 2PL model is computationally intensive and requires large sample sizes for accurate parameter estimates. If it is reasonable to assume that all items discriminate between respondents equally well, the 1PL model (simplified model which assumes the same discrimination parameter for all items in a test) may be a good alternative since it is less complex and requires fewer parameters to be estimated [48].

Moderated non-linear factor analysis (MNLFA) is a new, more general measurement model proposed by Bauer et al. [42]. It extends traditional factor analysis by allowing for non-linear relationships between observed variables and latent factors. Additionally, unlike IRT which is only able to detect and adjust for differential item functioning (DIF) for different discrete subgroups (e.g., sex, age group), MNLFA can account for moderating effects of categorical variables (e.g., sex, study membership) and continuous variables (e.g., age). Another strength of MNLFA compared with LFA and IRT is that MNLFA can accommodate mixed types of observed variables (e.g., continuous, binary and ordinal ones), while LFA and IRT require all items are of the same type. This flexibility makes MNLFA particularly useful for harmonizing data when variable types measuring the same construct vary greatly across studies and/or over time. MNLFA has been applied to harmonize mental health and substance use across longitudinal studies that used different assessment tools over time [6, 42, 49, 50]. While MNLFA is highly flexible, it requires expertise in advanced statistical modeling. To simplify the implementation of MNLFA in practice, the R package aMNLFA (Automated moderated non-linear factor analysis) has been developed [51]. Another obstacle for implementing MNLFA is that it is computationally demanding and requires large sample sizes to get stable parameter estimates.

### Methods to deal with systematically missing data in the pooled dataset

Another frequent issue in real world harmonization practices is systematically missingness that results from variables collected only in a subset of studies. Five articles [7, 8, 35, 40, 52] included in our review describe multiple imputation as a way to deal with systematically missing data. In the pooled dataset, values for missing variables in one study can be multiply imputed using information from variables overlapping across studies as well as other related variables. However, the proportion of systematically missing variables that can be validly handled through imputation in pooling studies has not yet been studied (e.g., based on simulation studies) and is hence unclear.

**Fig. 3:**
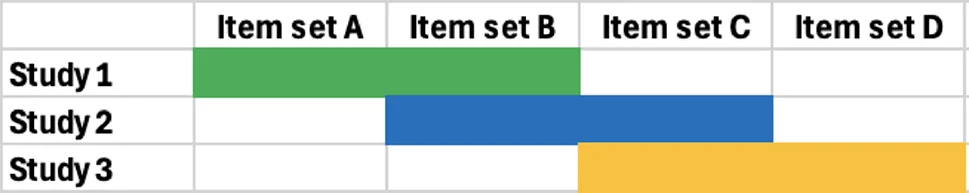
Schematic display how studies can be chained together by overlapping item pairs in latent variable analysis. Here overlapping items refer to items measuring the same latent construct with the same scales across studies. Study 1 and study 2 have overlapping item set B to derive comparable factors scores. Study 2 and study 3 have overlapping item set C to derive comparable factors scores. If study 1 and study 3 both have comparable scores with study 2, their scores are also comparable

Additionally, handling of systematically missingness in the pooled dataset mostly depends on the target variables required for the later analysis. In cases when latent construct variables are harmonized at the factor level and only factor scores are required for later analysis, the problem of systematically missing data at the item level (i.e., some items assessing the latent construct are only collected in a subset of studies) could be solved during the harmonization process. The measurement models can still be fitted even if no items are common to all studies. It only requires overlapping items which allow studies to be “chained” together and to derive comparable scores (see Fig. 3 for illustration). Similarly, the proportion score method could also handle the problem of systematically missing data at the item level by deriving a summary score.

### Proposed roadmap for selecting statistical method for data harmonization

**Fig. 4:**
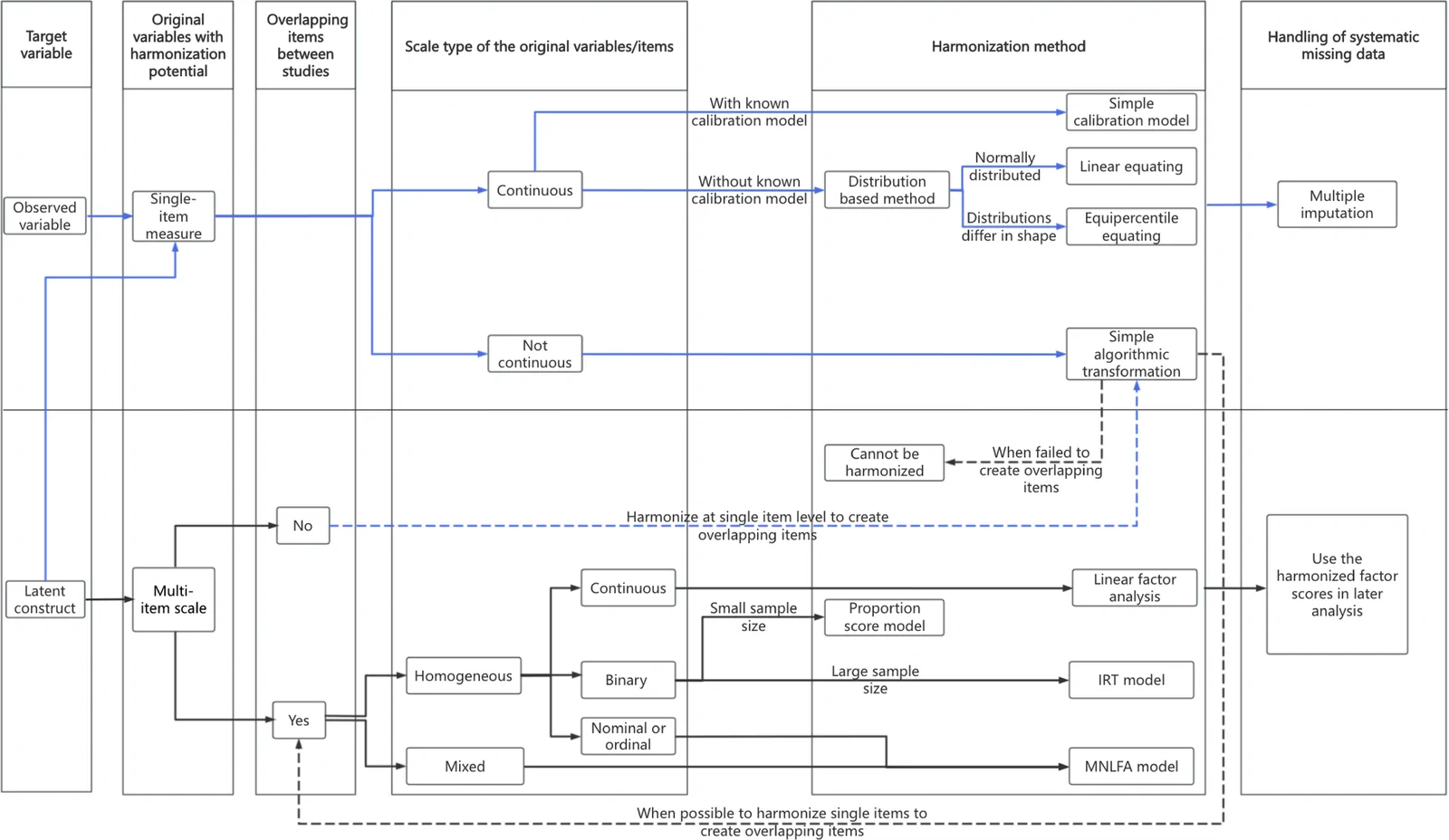
Roadmap for selecting statistical harmonization method for studies aiming to retrospectively harmonize data across multiple cohorts. The upper part of the roadmap with blue solid arrows shows the harmonization at the variable level when only single-item measures are available either for the observed target variables or for latent constructs. The lower part of the roadmap with black solid arrows summarizes the statistical harmonization methods for multi-item scales when the target variable is a latent construct

Our results have been summarized in a roadmap (see Fig. 4) that may guide researchers aiming to retrospectively harmonize data across multiple cohorts to select the most suitable statistical method for data harmonization.

When only single-item measures are available either for the observed target variables or for latent constructs, simple methods (i.e., simple algorithmic transformation and simple calibration) and distribution-based methods could be applied. Next, the scale type of the original variable needs to be considered. For continuous variables, the choice of a suitable harmonization method depends on specific characteristics of the original variables (e.g., whether a known calibration model exists, whether the distributions differ significantly in shape). If original variables are measured on binary, nominal or ordinal variables, simple algorithmic transformations are the method of choice. Systematically missing variables in the pooled dataset could be handled by multiple imputation.

When harmonizing a latent construct measured by multi-item scales, the proportion score method and more sophisticated latent variable models could be applied. Again, also the choice of latent variable models depends on the type of scale used for the items, as well as on the sample size and on the relationships between variables in the contributing studies. The LFA model and the IRT model are only appropriate if items are homogeneous with respect to scale type. If the items available for a given latent construct vary in scale type (i.e., mixed of continuous, ordinal, binary, count variables) across or within studies, MNLFA is the only option to be used. Additionally, as illustrated in Fig. 3, latent variable models require overlapping items which allow studies to be “chained” together and to derive comparable scores. If no common items for the same construct are available across studies, it is possible to apply simple algorithmic transformations to similar items and manually modify them to create overlapping items (as shown in dotted arrows in Fig. 4). Although overlapping items are not explicitly required by the proportion score model, it is typically used to derive a rough summary score from similar items. As mentioned above, in cases when latent construct variables are harmonized at the factor level and only factor scores are required for later analysis, the problem of systematically missing data at the item level can be solved during the harmonization process.

## Discussion

In this scoping review, we identified three types of statistical methods for data harmonization of longitudinal epidemiological data from 35 studies:1) distribution-based methods, 2) the proportion score model, and 3) latent variable models. The choice of the statistical method for data harmonization primarily depends on the nature of the target variable required for the later analysis (i.e., observed vs. latent), as well as on the scale type of the original variables. While all methods were identified from studies pooling longitudinal data, they are also applicable to cross-sectional data that lack the complexities introduced by the time dimension. Our roadmap comprehensively summarizes our findings and may guide researchers in selecting a suitable statistical method for data harmonization as well as in dealing with missing subsets of variables in future pooling initiatives.

In our review we focused on more sophisticated methods because inclusion of studies using simple methods would have led to too many hits without adding much useful information for our purpose. Nevertheless, simple methods such as algorithmic transformations and calibration are also integrated into our roadmap since they are commonly applied especially when harmonizing observed variables. Distribution-based methods are also frequently used when harmonizing observed variables with a continuous scale type. It would be also possible to apply simple methods or distribution-based methods to harmonize latent constructs measured by multi-item scales (e.g., applying simple methods to the selected item), but this typically leads to a loss of information. Bias may be introduced when the original observed indicators are measuring fundamentally different latent constructs. However, commonly applied latent variable models such as IRT and MNLFA often require large sample sizes. In situations where the sample size is small and a simple measure is sufficient, the proportion score model can be employed to generate summary scores. Additionally, latent variables models assume that observations are independent while this assumption is typically violated in longitudinal data containing repeated measurements on the same subjects. This problem is commonly solved by randomly selecting one observation per participant (despite the availability of repeated measures) to create a “calibration sample” for establishing the measurement properties of the latent construct. After fitting models to the calibration sample, item parameter estimates are obtained to generate factor scores for the full set of observations. It would be preferable to develop and apply new methods in future studies to account for the dependence structure in the data and utilize all available repeated measures to estimate item parameters. Another limitation of latent variable models is that at least some common items across studies are required to derive comparable scores. In situations where completely discrepant measurement instruments are administered across studies, Nichols et al. [61] suggested the so-called Linear Linking for Related Traits (LLRT) method and demonstrated it harmonizing cognitive performance across two (or more) groups based on cross-sectional data. Instead of leveraging information from common test items between cohorts, they leverage information from related constructs (and the common test items of this related construct). However, this solution relies upon strong assumptions and its applicability to longitudinal data requires further exploration.

When harmonizing data across studies, there is always a trade-off between accepting only precisely identical variables (e.g., exact same question in questionnaires) thereby limiting the number of incorporated studies/variables, versus accepting a certain degree of heterogeneity while incorporating more studies/variables. In most cases, transformation of raw data to harmonized data involves some loss or distortion of information since the information in each cohort is reduced to the lowest common denominator across cohorts [40]. Therefore, quality checks for the harmonized dataset would be desirable to decide whether the information loss during the harmonization process is acceptable. The Maelstrom Guidelines [8] as well as other domain specific data harmonization guidelines [62, 63] all empathize that both rigorous documentation and quality control are absolute essentials required to successfully harmonize various datasets. However, many pooling studies (125 out of 475 full text screened articles) do not provide detailed documentation about the harmonization process. Since rigorous documentation is essential for ensuring transparency and reproducibility of the harmonization process, future pooling studies are advised to document all important components of the harmonization process (e.g., original variables from contributing studies, the methods used to transform these variables, etc.). Methods and metrics for quality checks for the harmonized dataset are also rarely explicitly described and reported in pooling studies. Only 5 out of 25 pooling studies included in our review either conducted sensitivity analyses to show whether the results markedly differ under different harmonization decisions (e.g., when using richer data available in a smaller subset of cohorts compared to the lowest common denominator in all cohorts [6, 29, 38, 46]) or reported descriptive statistics for each harmonized variable and compared it with the initial cohort-specific data [33]. In the current literature, there is a lack of consensus on how to evaluate harmonization quality. The results from both, sensitivity analyses and descriptive statistics, are difficult to compare across studies and yet there are no established quality metrics. In future harmonization tasks, some measures of association between the generated harmonized variables and the original variables such as correlation coefficients suggested by some methodological papers and the Maelstrom guideline [39, 55, 64] could be reported to provide an indication on how well the derived variables reflect the original ones. When harmonizing observed variables, this procedure is quite straightforward, but problems arise when harmonizing latent constructs measured by multi-item scales as the derived factor variable is not available in the original datasets. In this case, correlations could be computed between the generated harmonized variables with related constructs (when available). If there is no related construct available in the original datasets, measurement invariance tests and model fit statistics could be considered as quality checks and should be reported in the respective studies.

From the included articles [33, 40, 43, 64], we have identified tools to facilitate the harmonization process, such as Opal [65] which enables the management of study data, ATHENA [66] and Usagi [67] that help with the standardization of vocabularies and the vocabulary mapping before the creation of the common data schema, and Mica [65] which could be used to build searchable web portals disseminating study and variable metadata. Specifically for the implementation of latent variables models, R packages such as aMNLFA [68] automate the steps of DIF detection and subsequent parameter estimation to simplify the scoring process. However, none of the pooling studies included in our results came up with automated procedures for deriving a harmonized dataset. Possible reasons may be that it is hard to evaluate the harmonization potential for variables in each study and decide on the processing rules without human assessment as this process requires a deep contextual understanding of the measured variables. Cohort profiles and data codebooks are often in descriptive text and are usually not structurally documented in similar format. Therefore, it is not easy to obtain comprehensive information from each study concerning cohort designs, participant follow-ups, and specific variables collected (e.g., whether blood glucose was measured in fasting status). The Metadata Annotation Workbench that was recently devised by the NFDI4Health (https://www.nfdi4health.de/en/) supports standardized metadata annotation and fosters the interoperability of data collection instruments for epidemiological studies [69]. Another step towards automated data harmonization is the Harmony tool that uses natural language processing to match individual questions based on their semantic content [70]. These tools help to identify harmonizable variables based on input content. The decisions about how to process the variables into a common format remain with the researchers. Current versions of these tools are designed to match the tabular format metadata and do not comment on how to handle large bodies of text such as cohort profiles. These tools have great potential and would benefit from further enhancements to fully automate the harmonization process.

There are no standard keywords or mesh terms used in bibliographic databases to identify literature describing statistical methods or algorithms for harmonizing longitudinal epidemiological data. The systematic search conducted in our review included terms related to “data harmonization” or “data pooling”, and terms related to “longitudinal data”. We did not constrain our searches by keywords related to “epidemiology” or any specific statistical methods in order not to restrict our search to a specific discipline, so also machine learning-based methods could be included, but none were identified in our search. A possible explanation could be that machine learning-based methods are mainly applied in the context of more complex data types, such as imaging data, which falls outside the scope of our review. Furthermore, recently emerging machine learning-based methods may not yet be indexed in bibliographic databases. New developments in this field are often found in alternative sources, such as Consensus.app, Google, and preprint repositories like arXiv. We cannot preclude that there are additional statistical methods or algorithms based on machine learning for data harmonization beyond those discussed in this review which we may have missed by our search strategy.

## Conclusion

This scoping review provides an overview of different statistical methods used for retrospective data harmonization of longitudinal epidemiological studies. The proposed roadmap may guide researchers aiming to conduct a pooling study on which method to use in a specific harmonization setting and on how to handle systematically missing data collected only in a subset of studies. Our results also highlight the necessity of rigorous documentation in harmonization projects. In addition, researchers should perform quality checks to test whether the generated variables adequately reflect the original ones. While first steps are taken, novel automated tools/procedures should be developed to facilitate the demanding harmonization process of epidemiological data in future studies.

## Supplementary Information

Below is the link to the electronic supplementary material.

Supplementary Material 1
