# Supplementary material for: Statistical methods for retrospective harmonization of longitudinal epidemiological data: a scoping review
Source: Eur J Epidemiol. 2026 May 28;41(8):935–47. doi: 10.1007/s10654-026-01404-3 (PMC13612613; doi:10.1007/s10654-026-01404-3)
Supplement: Supplementary file 1 [file 10654_2026_1404_MOESM1_ESM.docx]

**Supplementary File: A scoping review of statistical methods for retrospective harmonization of longitudinal epidemiological data**

[Appendix A: Search Strategy	2](#_Toc181567774)

[Database: PUBMED	2](#_Toc181567775)

[Database: Web of Science	3](#_Toc181567776)

[Database: IEEE	4](#_Toc181567777)

[Appendix B: Eligibility criteria for scoping review	5](#_Toc181567778)

[Appendix C: Data extraction form of methodological papers	6](#_Toc181567779)

[Appendix D: Data extraction form of pooling studies	8](#_Toc181567780)

[Appendix E: Application examples of identified harmonization method	21](#_Toc181567781)

Figure 1 Flowchart depicting the selection process

# Appendix A: Search Strategy

## Database: PUBMED

**Total hits**: 859

| **Query** | **Results** |
| --- | --- |
| **Concept block defined by inclusion criteria (1): data harmonisation**  (((((((("data harmonization") OR ("data harmonisation")) OR ("retrospective harmonization")) OR ("retrospective harmonisation")) OR ((("harmonized data") OR ("harmonised data")) OR ("harmonized analysis"))) OR (("data integration") OR ("integrative data analysis"))) OR ("individual participant data meta-analysis")) OR ((("individual-level data") OR ("individual participant data")) AND ((((("data pooling") OR ("pooled data")) OR ("pooled analysis")) OR ("combine data")) OR ("data combination")))) OR ((("multi-study") OR ("multi-cohort")) OR ("cross-cohort")) | 8493 |
| **Concept block defined by inclusion criteria (2): longitudinal data**  (cohort studies[MeSH Terms]) OR ((((("longitudinal data") OR ("panel data")) OR ("longitudinal study")) OR ("longitudinal studies")) OR ("longitudinal measures")) | 2602356 |
| **Concept block defined by inclusion criteria (1): + Concept block defined by inclusion criteria (2):**  ((((((((("data harmonization") OR ("data harmonisation")) OR ("retrospective harmonization")) OR ("retrospective harmonisation")) OR ((("harmonized data") OR ("harmonised data")) OR ("harmonized analysis"))) OR (("data integration") OR ("integrative data analysis"))) OR ("individual participant data meta-analysis")) OR ((("individual-level data") OR ("individual participant data")) AND ((((("data pooling") OR ("pooled data")) OR ("pooled analysis")) OR ("combine data")) OR ("data combination")))) OR ((("multi-study") OR ("multi-cohort")) OR ("cross-cohort"))) AND ((cohort studies[MeSH Terms]) OR ((((("longitudinal data") OR ("panel data")) OR ("longitudinal study")) OR ("longitudinal studies")) OR ("longitudinal measures"))) | 986 |
| **Filter to exclude highly targeted towards the integration of specific datasets:**  ((((((((((((((("data harmonization") OR ("data harmonisation")) OR ("retrospective harmonization")) OR ("retrospective harmonisation")) OR ((("harmonized data") OR ("harmonised data")) OR ("harmonized analysis"))) OR (("data integration") OR ("integrative data analysis"))) OR ("individual participant data meta-analysis")) OR ((("individual-level data") OR ("individual participant data")) AND ((((("data pooling") OR ("pooled data")) OR ("pooled analysis")) OR ("combine data")) OR ("data combination")))) OR ((("multi-study") OR ("multi-cohort")) OR ("cross-cohort"))) AND ((cohort studies[MeSH Terms]) OR ((((("longitudinal data") OR ("panel data")) OR ("longitudinal study")) OR ("longitudinal studies")) OR ("longitudinal measures")))) NOT (MRI[Title/Abstract])) NOT (sequencing[Title/Abstract])) NOT (radiomics[Title/Abstract])) NOT (omics[Title/Abstract])) NOT (GWAS[Title/Abstract])) NOT (imaging[Title/Abstract]) | 859 |

## Database: Web of Science

**Total hits: 592**

| **#** | **Query** | **Results** |
| --- | --- | --- |
| 1 | ALL=("individual participant data meta-analysis") | 740 |
| 2 | ALL=("multi-cohort") | 660 |
| 3 | (#2) OR ALL=("multi-study") | 986 |
| 4 | (ALL=("data harmonization")) OR ALL=("data harmonisation") | 736 |
| 5 | (ALL=("harmonized data")) OR ALL=("harmonised data") | 402 |
| 6 | (ALL=("data integration")) OR ALL=("integrative data analysis") | 9836 |
| 7 | (ALL=("harmonized analysis")) OR ALL=("harmonised analysis") | 22 |
| 8 | (ALL=("retrospective harmonization")) OR ALL=("retrospective harmonisation") | 14 |
| 9 | ((ALL=("data pooling")) OR ALL=("pooled data")) OR ALL=("pooled analysis") | 31988 |
| 10 | (ALL=("data combination")) OR ALL=("combine data") | 1842 |
| 11 | #9 OR #10 | 33807 |
| 12 | (((ALL=("individual-level data")) OR ALL=("individual patient data")) OR ALL=(IPD)) OR ALL=("individual participant data") | 14348 |
| 13 | #11 AND #12 | 920 |
| 14 | #1 OR #3 OR #4 OR #5 OR #6 OR #7 OR #8 OR #13 | 13485 |
| 15 | (((((((ALL=("cohort study"))) OR ALL=("longitudinal data")) OR ALL=("panel data")) OR ALL=("longitudinal study")) OR ALL=("longitudinal studies")) OR ALL=("longitudinal measures")) OR ALL=("cohort studies") | 440581 |
| 16 | #14 AND #15 | 673 |
| 17 | ((((((#16) NOT TS=(MRI)) NOT TS=(omics)) NOT TS=(sequencing)) NOT TS=(radiomics)) NOT TS=(imaging)) NOT TS=(GWAS) and English (Languages) and 2023 or 2022 or 2021 or 2020 or 2019 or 2018 or 2017 or 2016 or 2015 or 2014 or 2013 or 2012 or 2011 or 2010 or 2009 or 2008 or 2007 or 2006 or 2005 or 2004 or 2003 or 2002 or 2000 (Publication Years) | 592 |

## Database: IEEE

**Total hits:** 134

(((Full Text Only:cohort study) OR (Full Text Only:longitudinal data) OR (Full Text Only:longitudinal study) refined by:Year:2000-2024 )) AND ((Full Text Only:data harmonization) OR (Full Text Only:data harmonisation) OR (Full Text Only:retrospective harmonization) OR (Full Text Only:retrospective harmonisation) OR (Full Text Only:harmonized data) OR (Full Text Only:harmonised data) OR (Full Text Only:harmonized analysis) OR (Full Text Only:data integration) OR (Full Text Only:integrative data analysis) OR (Full Text Only:multi-study) OR (Full Text Only:multi-cohort) refined by:Year:2000-2024 )

# Appendix B: Eligibility criteria for scoping review

|  | **Inclusion** |
| --- | --- |
| Language | English only |
| Species | Human Only |
| Time Frame | From January 1, 2000, to December 25, 2023 |
| Main content | Describe or apply methodology for retrospectively harmonizing longitudinal data at the individual level |

|  | **Exclusion** |
| --- | --- |
| Adaptability to epidemiological data | Studies on methods that are highly targeted towards the integration of specific datasets (e.g. omics and imaging datasets) |
|  | Studies not health-oriented (e.g. GIS data from ecology) |
| Methodology for retrospectively harmonizing longitudinal data at the individual level | Studies describing/using data linkage methods |
|  | Studies that do not pool data at individual level (e.g. two-stage meta-analysis) |
|  | Studies pooling cross-sectional data |
|  | Studies on pipelines and tools designed to assist data collectors in obtaining standardized data while collecting new data (i.e. studies on prospective data harmonization) |
|  | Studies that do not provide information about the harmonization process |
| Focus on more sophisticated statistical methods | Studies that use only simple algorithmic transformation or simple calibration method |

# Appendix C: Data extraction form of methodological papers

Table 1 Descriptive characteristics of included methodological papers summarizing statistical harmonization methods without an empirical example for illustration

| **No** | **Article title** | **Author** | **Year** | **Statistical harmonization method** | **Tools/procedures to facilitate harmonization** | **Quality checks for harmonized dataset** | **Methods to deal with missing data** |
| --- | --- | --- | --- | --- | --- | --- | --- |
| 1) | Integrative data analysis through coordination of measurement and analysis protocol across independent longitudinal studies | Hofer et al. | 2009 | Standardization, proportion score model, Item response theory (IRT) model | NA | Using IRT model to identify plausible differential item functioning (not explicitly mentioned as quality checks for harmonization process) | NA |
| 2） | Maelstrom Research guidelines for rigorous retrospective data harmonization | Fortier et al. | 2016 | Standardization, latent variable model | NA | Descriptive statistics to evaluate consistency of the harmonized data across studies | Multiple imputation model |
| 3） | Advanced analytical methodologies for measuring healthy ageing and its determinants, using factor analysis and machine learning techniques: the ATHLOS project | Caballero et al. | 2017 | Bayesian multilevel IRT model | NA | The degree of relationship between the metric generated and a simple sum of the items was assessed by calculating correlation coefficient | NA |
| 4) | Advancing the study of adolescent substance use through the use of integrative data analysis | Curran et al. | 2018 | Moderated non-linear factor analysis (MNLFA) | NA | Using MNLFA to identify plausible differential item functioning (not explicitly mentioned as quality checks for harmonization process) | Although several items are only available in a subset of contributing studies, entire set of items can be retained in the factor model given that that absent items are treated as missing at random |
| 5） | Cohort harmonization and integrative analysis from a biomedical engineering perspective | Kourou et al. | 2019 | Latent variable model (Linear factor analysis, IRT model) | DataSHaPER: enables the generation of a list of core variables among participating studies through a template that defines common format measures; Opal: enables the management of study data; ATHENA and Usagi: help with the standardization of vocabularies and the vocabulary mapping, respectively, before the creation of the common data schema | Correlations were computed between the generated harmonized variables (latent factor) and similar constructs from previous studies | NA |
| 6） | Life course of retrospective harmonization initiatives: key elements to consider | Fortier et al. | 2022 | Standardization, latent variable model | NA | Descriptive statistics to evaluate the consistency across cohort studies; testing for a statistical effect of study-specific question formats on the harmonized data generated) | Multiple imputation model |
| 7) | Harmonizing single-question instruments for latent constructs with equating using political interest as an example | Singh et al. | 2022 | Linear equating, equipercentile equating | NA | Correlations were computed between the generated harmonized variables with related constructs | NA |
| 8） | Retrospective psychometrics and effect heterogeneity in integrated data analysis: commentary on the special issue | Howe et al. | 2023 | Moderated non-linear factor analysis (MNLFA) | An automated R package: automated moderated nonlinear factor analysis (aMNLFA) to simplify the implementation of MNLFA scoring | Using MNLFA to identify plausible differential item functioning (not explicitly mentioned as quality checks for harmonization process) | NA |
| 9） | Harmonizing ethno-regionally diverse datasets to advance the global epidemiology of dementia | Lipnicki et al. | 2023 | Standardization, latent variable modelling (LFA, IRT, MNLFA), multiple imputation | DataSHaPER and BioSHaRE: structured framework for harmonizing existing studies | Using IRT model to identify plausible differential item functioning (not explicitly mentioned as quality checks for harmonization process) | Multiple imputation |
| 10) | Advancing intervention and prevention research for behavioral health problems through data synthesis | Pearson et al. | 2023 | MNFLA | NA | NA | NA |

# Appendix D: Data extraction form of pooling studies

Table 2 Descriptive characteristics of included 1) pooling studies and 2) methodological papers proposing/summarizing statistical harmonization methods with an empirical example for illustration

| **No** | **Article title** | **Author** | **Year** | **Purpose of pooling** | **No. of studies pooled** | **Pooled sample size** | **Age span in pooled dataset** | **Harmonized variable** | **Statistical Harmonization method** | **Tools to facilitate harmonization** | **Quality checks for harmonized dataset** | **Methods to deal with missing data** |
| --- | --- | --- | --- | --- | --- | --- | --- | --- | --- | --- | --- | --- |
| 1) | Disaggregating the distal, proximal, and time-varying effects of parent alcoholism on children's internalizing symptoms | Hussong et al. | 2008 | To increase sample size and cover longer observation period to examine effects differentially focus on inter-individual and intra-individual differences and on influences that largely precede versus occur within the assessment period | 2 | 2418 | 2-17 | Child internalizing symptoms | Two-parameter logistic IRT model (2 PL) | NA | Test for DIF in age child gender, and study sequentially; derive separate item parameter estimates as a function of age or gender so that subsequent scoring would account for DIF | For systematic missing data in items assessing internalizing symptoms: compute IRT scores for later analysis; for missing data in covariates: multiple imputation |
| 2) | Pooling data from multiple longitudinal studies: the role of item response theory in integrative data analysis | Curran et al. | 2008 | increased statistical power, greater sample heterogeneity in important subject demographics, the broader psychometric assessment of constructs, and the ability to estimate a variety of models that would not be possible within any single data set, to study a longer maturational period than was assessed within any single investigation | 3 | 1827 | 10-33 | Internalizing symptomatology | Proportion score model, two- parameter logistic IRT model (2 PL) | NA | Graphically present the item parameters estimates from the IRT model to detect possible differential item functioning (DIF) ; use the IRT model to conduct tests of DIF | For systematic missing data in items assessing internalizing symptoms: compute IRT scores for later analysis |
| 3) | Psychometric approaches for developing commensurate measures across independent studies: traditional and new models | Bauer et al. | 2009 | Increased age span in the harmonized data permits to examine consistency in the pattern of change identified in different development periods and increased sample size augments the ability to detect effects in different developmental periods | 2 | 3602 | 10-22 | Alcohol involvement | Linear factor analysis, IRT model, MNLFA | NA | Graphical analysis to check whether all items are reflections of a single underlying factor; conduct DIF tests to formally evaluate measurement equivalence over studies; boxplots of estimated factor scores obtained at each age for the two studies, shown in relation to the model implied means and ± two standard deviations | For systematic missing non-common items (due to the use of different measurement instruments across studies): the inclusion of non-common items is not problematic when fitting the measurement model |
| 4) | Parent alcoholism impacts the severity and timing of children's externalizing symptoms | Hussong et al. | 2010 | Greater power to test interactions; abie to test the hypotheses of interest over a longer developmental age range than is possible in any one of the two studies alone | 2 | 2418 | 2-17 | Child externalizing symptoms | Two-parameter logistic IRT model (2 PL) | IRTLRDIF software automates the steps of DIF detection and subsequent IRT parameter estimation | Tested for DIF in age child gender, and study sequentially; derive separate item parameter estimates as a function of age or gender so that subsequent scoring would account for DIF | For systematic missing data in items assessing externalizing symptoms: compute IRT scores for later analysis; missing data in covariates: multiple imputation |
| 5) | Understanding ageing in older Australians: The contribution of the Dynamic Analyses to Optimise Ageing (DYNOPTA) project to the evidence base and policy | Anstey et al. | 2011 | To investigate possible cohort differences in change; to extend age range extends allowing us to examine health trajectories across a braoder range | 9 | 50652 | 45-106 | Depression | Latent variable model | NA | NA | Multiple imputation |
| 6) | Integrative data analysis in clinical psychology research | Hussong et al. | 2013 | Larger sample size, longer periods of development than captured in any one of our contributing studies, ability to compare subgroups of children with small representation in the contributing studies | 3 | 2006 | 2-40 | Internalizing behavior | MNLFA | NA | Sensitivity analysis: repeat the measurement building process with a second calibration sample drawn from the pooled sample and found that scores were highly correlated across the two calibration samples | For systematic missing data in items assessing internalizing behavior: compute MNLFA scores for later analysis |
| 7) | Data integration approaches for longitudinal growth modeling | Katerina et al. | 2017 | To fit complex model that is not estimable in any of the individual datasets; to create a more heterogeneous sample, which can increase generalizability of results | 6 | 10459 | 0-22 | Mathematics ability | One-parameter logistic item response theory (IRT) model (1PL) | NA | NA | For systematic missing data in items assessing mathematics ability: compute IRT scores for later analysis |
| 8) | Modeling pathways of character development across the first three decades of life: an application of integrative data analysis techniques to understanding the development of hopeful future expectations | Callina et al. | 2017 | To increase power and cover relatively longer time spans | 4 | 20736 | 7-26 | Hopeful future expectations | Confirmatory factor analysis (CFA) | NA | Employed CFA to test measurement invariance across different age groups within each study and across studies: model fit statistics (RMSEA: Root Mean Square Error of Approximation, CFI: Comparative Fit Index, TLI: Tucker-Lewis Index) suggested acceptable fit for most models in both the longitudinal and between study invariance tests | To compute the hopeful future expectations scale scores, they simply used the average of hopeful future expectations items that met longitudinal and between-study invariance for each set of studies, which do not require items are available in all studies |
| 9) | Cohort profile: the MULTI sTUdy Diabetes rEsearch (MULTITUDE) consortium | Pino et al. | 2018 | To have large sample size and generally long follow-up period that facilitates examination of T2DM risk and outcomes across the life course | 17 | 135156 | 3-88 | Blood cholesterol concentration | Standardization model | NA | NA | NA |
| 10) | Development of a clinical prediction model for the onset of functional decline in people aged 65-75 years: pooled analysis of four European cohort studies | Jonkman et al. | 2019 | To increase sample size; to assess heterogeneity of findings across the cohorts and evaluate the external validity of the model | 4 | 2560 | 65-75 | Gait speed | Z-scores | NA | NA | Systematic missing variable: NA; missing values on predictors: multiple imputation within each cohort |
| 11) | A latent variable modelling approach for the pooled analysis of individual participant data on the association between depression and chlamydia infection in adolescence and young adulthood in the UK | Koukounari et al. | 2019 | Pooling two studies to cover the broader developmental period which would not have been possible if the analysis had been conducted separately for each of the two studies | 2 | 12271 | 10-24 | Depression score | Graded response IRT model | NA | Tested for measurement invariance by study in the items within IRT model; cross-validated with a new randomly selected calibration sample from the two studies; conducted sensitivity analysis by including only ALSPAC data; outlier detection analyses using graphical representations of the scores as a function of specific person covariates | For systematic missing data in items assessing depression: compute IRT scores for later analysis |
| 12) | Congruent relations between perceived neighborhood social cohesion and depressive symptoms among older European adults: An East-West analysis | Ruiz et al. | 2019 | To increase sample size and sample heterogeneity to allow cross-cultural comparisons that reflect the region's diversity | 2 | 26081 | 45 + years | Depressive symptoms, perceived neighborhood social cohesion | Proportion score model, country-specific tertiles | NA | Restricted the models to participants without depressive symptoms at baseline to confirm that the longitudinal relations hypothesized by the model were not biased by depressed individuals at baseline | Systematic missing variables (different item versions were implemented at follow-up, some items are eliminated): use version-specific cut-off to categorize depressive disorders probable cases; missing data: multiple imputation |
| 13) | Brazilian maternal and child nutrition consortium: establishment, data harmonization and basic characteristics | Carrilho et al. | 2020 | To increase sample size and improve the statistical power of the analyses and thus strengthen the robustness and relevance of the results; to maximize the existing resources; to answer questions that the individual studies could not answer themselves; to offer an opportunity for collaboration among researchers from different institutions and areas | 21 | 17344 | 18-47 | Length at birth | Z-scores | NA | NA | NA |
| 14) | Low protein intake, physical activity, and physical function in European and North American community-dwelling older adults: a pooled analysis of four longitudinal aging cohorts | Mendonça et al. | 2021 | To increase sample size and test for interactions that could not be tested otherwise | 4 | 3075 | 70-79 | Energy intake, walking speed, physical activity scores, cognitive function | Z-scores and categorization based on cohort-specific tertiles | NA | NA | NA |
| 15) | Multiple social roles in early adulthood and later mental health in different labour market contexts | Engels et al. | 2021 | The use of multiple sources of data and the large sample size making the findings applicable to a much wider population, including the majority of countries in the European Union | 2 | 43502 | 25-40 | Depression | Z-scores | NA | NA | NA |
| 16) | Overview of retrospective data harmonization in the MINDMAP project: process and results | Wey et al. | 2021 | To provide statistical power required to investigate direct and interactive effects of social, environmental and lifestyle determinants of mental health outcomes across different cities | 6 | 220621 | 14-102 | Cognitive measures | Standardization | Opal: a database application for storing, managing and transforming study data from multiple sources; Mica: used to build searchable web portals disseminating study and variable metadata | To explore harmonized data content within each subpopulation, univariate distributions were generated for each DataSchema variable and reviewed for correct participant numbers, distributions and missing values, compared to the initial cohort-specific data provided | NA |
| 17) | Abnormal cognitive aging in people with HIV: evidence from data integration between two countries' cohort studies | Aung et al. | 2022 | To increase sample size and improve the generalizability of research output as the sample will become more heterogeneous | 2 | 1026 | Age range not reported (mean age = 45.22, SD = 8.86) | Neuropsychological test score | Demographically corrected T scores | NA | NA | NA |
| 18) | Development of the ADFICE_IT models for predicting falls and recurrent falls in community-dwelling older adults: pooled analyses of European cohorts with special attention to medication | Van De Loo et al. | 2022 | To improve predictive performance by increasing sample size and make the prediction models generalizable to a wider range of populations | 3 | 5722 | 65-98 | Grip strength, verbal fluency, depression score, physical activity, functional limitations, processing speed, and gait speed | Z-scores | NA | NA | Multiple imputation |
| 19) | Impact of taxane-based chemotherapy among older women with breast cancer on cognition and quality of life: a longitudinal pooled analysis | Small et al. | 2022 | To increase sample size | 2 | 167 | 60-70 | Objective cognitive performance | Z-scores | NA | NA | NA |
| 20) | Overview of CAPICE—Childhood and Adolescence Psychopathology: unravelling the complex etiology by a large Interdisciplinary Collaboration in Europe—an EU Marie Skłodowska‑Curie International Training Network | Rajula et al. | 2022 | To enable a joint analysis of data over cohorts | 8 | 73714 | 3-18 | Internalizing and ADHD symptoms | IRT model | A searchable data catalogue is created: a search engine that returns items including the searched term as well as related terms by text mining of available data documentation to identify relationships between words | NA | For systematic missing items assessing internalizing and ADHD symptoms: compute IRT scores for later analysis |
| 21) | Developmental Models of Depression, Externalizing Problems, and Self-regulatory Processes: Integrated Data Analysis Across Four Longitudinal Studies of Youth | Magee et al. | 2023 | To increase statistical power and provide a deeper understanding of how these constructs develop over time | 4 | 4773 | 10.5-20 | Depression severity and externalizing problems | MNLFA | Moderated nonlinear factor analysis conducted with an automated R package (aMNLFA) | Conduct tests to examine invariance across covariates with respect to factor means, variances, and item intercepts/factor loadings to obtain valid item parameter estimates adjusting for DIF | MNLFA model creates comparable estimates of constructs across datasets that contain at least some common items, coupled with unique items that appear in only one or a subset of datasets |
| 22) | Informing Harmonization Decisions in Integrative Data Analysis: Exploring the Measurement Multiverse | Cole et al. | 2023 | To increase power, particularly for low base rate or small sub-group analyses | 3 | 2245 | 13-18 | Delinquent behaviour | MNLFA | The automated MNLFA (aMNLFA) procedure and accompanying R package aMNLFA | Conducted iterative, item-wise DIF tests in MNLFA | Systematic missing data in items assessing psychological distress: MNLFA scores |
| 23) | Long-term psychological distress trajectories and the COVID-19 pandemic in three British birth cohorts: A multi-cohort study | Moreno-Agostino et al. | 2023 | To cover longer observation period | 3 | 16389 | 23-75 | Psychological distress | IRT model | NA | Conducted sensitivity checks: used alternative psychological distress operationalisations, in addition to the main operationalisation as a factor score | Systematic missing data in items assessing psychological distress:IRT scores |
| 24) | Sex differences in cognitive aging and the role of socioeconomic status: Evidence from multi-cohort studies | Jin et al. | 2023 | To increase sample size and improve the generalizability of their findings given that patterns could be different between countries with different levels of development | 4 | 61019 | 50-100 | Cognitive function scores | Z-scores | NA | NA | NA |
| 25) | The Role of Smoking, Obesity, and Physical Inactivity in Cognitive Performance and Decline: A Multicohort Study | Hong et al. | 2023 | To increase sample size and extend observation periods to examine the role of healthy lifestyles in the performance and its decline rate of specific cognitive domains by birth cohort | 4 | 52233 | 50-100 | Cognitive function | Z-scores | NA | Conducted sensitivity analyses: separately plotting the cognitive decline rate for participants in each study | Participants with missing information on cognitions or lacking data on lifestyles, and participants with missing information on covariates at the baseline, were excluded |

# Appendix E: Application examples of identified harmonization method

| **Harmonization method** | | **Application example** |
| --- | --- | --- |
| Distribution based method | Linear equating | **Latent construct (composite score)**: Hong et al [1] combined data from 4 prospective cohort studies (China Health and Retirement Longitudinal Study, English Longitudinal Study on Ageing, Health and Retirement Study, and Survey of Health, Ageing and Retirement in Europe) to detect the role of smoking, obesity, and physical inactivity in cognitive function domains (epidemic memory, working memory, and time orientation). These 4 studies have similar cognition measures of episodic memory, working memory, and time orientation. All measures were standardized to facilitate comparisons, using the z-score of each cognitive domain, calculated by subtracting the mean and dividing by the standard deviation (SD) across all participants.  **Observed variable**: Mendonça et al [2] pooled data from cohorts in the PROMISS (PRevention Of Malnutrition In Senior Subjects in the European Union) consortium to investigate the longitudinal relation between protein intake and physical function, and the interaction with physical activity. Because the methods to assess energy intake and walking speed were different between cohorts, these were transformed into cohort-specific z-scores [z = (x − μ)/σ)] to facilitate comparisons. |
|  | Equipercentile equating | **Categorization based on cohort-specific tertiles**: Mendonça et al [2] pooled data from cohorts in the PROMISS (PRevention Of Malnutrition In Senior Subjects in the European Union) consortium to investigate the longitudinal relation between protein intake and physical function, and the interaction with physical activity (PA). PA was transformed into cohort-specific tertiles (categorized as low, medium, and high) at baseline, which were used to categorize the PA score for subsequent waves. |
| Others | Proportion score model | **Scale version-specific cut-off based on proportion score**: Ruiz et al [3] pooled data from the English Longitudinal Study of Ageing (ELSA), and the Health, Alcohol and Psychosocial factors In Eastern Europe (HAPIEE) studies to compare the prospective association between perceived neighbourhood social cohesion and depressive symptoms among older adults. The Center for Epidemiological Depression (CES-D) scale was used to measure depressive symptoms in both studies. Different scale versions were implemented with yes/no response options at follow-up, respectively in ELSA and HAPIEE. To account for baseline risk of probable depression in each study, ELSA participants with CES-D 8 scores of 3 ≥ were classified as probable cases. As HAPIEE employed the original CES-D 20 scale at baseline, HAPIEE participants with CES-D 20 scores of 16 ≥ were categorised as probable cases. |
| Latent variable model | Linear factor analysis (LFA) | Callina et al[4] pooled raw data from four studies evaluating youth character strengths in different settings to fit latent growth curve models of hopeful future expectations from participants aged 7 through 26 years. The prompt of the items related to hopeful future expectations was formatted differently for the younger participants in one contributing study. Confirmatory factor analysis is employed to test whether a construct and its items are comparable across ages within study and then between studies. A single latent factor is posited to underlie a set of observed, continuous indicator variables(i.e., items). The model is specified as:  $y_{ij}=\nu_{j}+\lambda_{j}\eta_{i}+\epsilon_{ij}$  where j indexes the item (j = 1, 2,..., p) and i indexes the person (from i = 1, 2,..., N). The relationship between each observed item y and the latent factor η is modeled by a simple linear regression with the intercept and slope (loading) parameters indicated by ν and λ. ν and λ are jointly referred to as item parameters. Additionally, the factor and residuals are normally and independently distributed as η_j_ ∼ N(α, ψ) and$\epsilon_{ij}\sim N\left( 0,\sigma_{i}^{2} \right)$. Measurement invariance was established across different age groups within each study data set first, and then they tested for invariance across studies within the pooled data set. The longitudinal invariance was evaluated by testing whether item parameters are the same across time. The between-study invariance proceeded similarly as the longitudinal invariance. Finally, the average of items that met longitudinal and between-study invariance are computed and used as the hopeful future expectations scale scores in later analysis. |
|  | Item response theory (IRT) model | **One-parameter logistic IRT model (1PL)**: Marcoulides et al[5] combined six longitudinal studies of mathematics ability in children with a goal of examining individual changes in mathematics ability and to determine differences in the trajectories based on sex and socioeconomic status. These studies applied different versions of the Applied Problems (AP) subtest (WJ-R and WJ-III) to assess mathematics ability in children. The AP test from the WJ-R contains 60 items, the AP test from the WJ-III contains 63 items, and the two versions share 39 items. To address the issue of different versions of the AP subtest, they fitted 1PL model to the item-level data to estimate the mathematics ability scores that were not dependent on the version of the subtest. The 1PL is specified as:  $P\left( y_{ij}=1 \mid\theta_{i},\beta_{j} \right)=\frac{\exp\left( \theta_{i}-\beta_{j} \right)}{1+\exp\left( \theta_{i}-\beta_{j} \right)}$  where $P\left( y_{ij}=1 \mid\theta_{i},\beta_{j} \right)$is the probability of getting item j in the AP subtest correct given person i’s mathematic ability level θ_i_, and item j’s difficulty β_j_. The difficulty parameter of a given item (i.e., difficulty β) represents the value on θ that is needed to have a probability of 0.50 of endorsing a given item correctly. Larger β of an item reflect the item is more difficult because higher mathematic ability level is needed to obtain a probability of 0.50 to correctly respond to the item. In this specification, the 39 items that were common to both versions have the same item parameters, which places the mathematic ability level on the same scale regardless of the version of AP subtest. The estimates of the mathematic ability scores were then used as observed data in subsequent analyses.  **Two-parameter logistic IRT model (2PL)**: Curran et al [6] considered data drawn from three existing longitudinal studies to evaluate individual variability in developmental trajectories of internalizing symptomatology spanning from childhood through adolescence and into young adulthood. These studies administered 27 dichotomous items from two different scales: the Brief Symptom Inventory (BSI) and the Child Behavior Checklist (CBCL). Of the total 27 items administered in all three studies, only six common items were identical between the BSI and CBCL (i.e., precisely the same item content appeared on both scales). To address the issue of different measures of internalizing symptomatology in three studies, they fitted 2PL model to the item-level data to estimate symptomatology scores that were not dependent on the administered scales. The 2PL is specified as:  $P\left( y_{in}=1 \mid\theta_{n},{\alpha_{j},\beta}_{j} \right)=\frac{1}{1+e^{-1.7\alpha_{j}\left( \theta_{i}-\beta_{j} \right)}}$  where $P\left( y_{in}=1 \mid\theta_{n},{\alpha_{j},\beta}_{j} \right)$ is the probability of positively endorsed item j given person i’s internalizing symptomatology level θ_i_, and item j’s difficulty β_j_ and discrimination $\alpha_{j}.$Similar to 1PL, larger values of β reflect that higher levels of θ are needed to positively endorse the item. In addition, the discrimination parameter of the item j (i.e., $\alpha_{j}$) would represent the strength of the relation between the item j and the internalizing symptomatology level θ. Larger value of α for a given item reflect the given item is able to better discriminate among individuals. They then used the 2PL model to conduct tests of differential item functioning, which is a formal inferential test of the equality of the difficulty and discrimination parameters for each item across one or more discrete groups (e.g., study membership, age group). For items reflect DIF, unique item parameters were estimated within each age/study group. After completing DIF testing, the item parameters were used to calculate person-and time-specific estimates of the internalizing symptomatology level. |
|  | Moderated nonlinear factor analysis (MNFLA) | A detailed description of MNLFA could be found in [7] and a brief summary is provided as follows.  The LFA and 2PL model are subsumed under the generalized linear factor analysis (GLFA) model. The GLFA model is written as:  $g_{j}\left( \mu_{ij} \right)=\nu_{j}+\lambda_{j}\eta_{i}$  where μ_ij_ is the expected value of item j for person i. Like the LFA model, ν_j_ and λ_j_ represent the intercept and factor loading for the item jand η_i_ is the latent factor, assumed to be normally distributed as η_i_∼ N(α, ψ). In relation to a typical generalized linear model (GLM), ν_j_ + λ_j_η_i_ takes on the role of the linear predictor, and g_j_ is the link function. When all items are continuous, we could choose the identity link, defined as g_j_(μ_ij_) = μ_ij_, to reproduce the LFA model. When all items are binary, we could choose the logit link function, defined as g_j_(μ_ij_) = ln [μ_ij_/(1 – μ_ij_)], to reproduce the 2PL model.  The MNLFA model extends the GLFA model by 1) removing the restriction that the response distributions for the items belong to the exponential family; 2) allowing the items to be modeled as nonlinear functions of the item parameters and the latent factor; 3)allowing the parameters of the model to be moderated by observed predictors, including both discrete (e.g., study) and continuous (e.g., age) variables.  Bauer et al[7] combined data from two independent longitudinal studies from age 10 to 22 to investigate the changes of involvement with alcohol when children enter adolescence and then progress to adulthood. The response scales of alcohol involvement items differed within and between studies. The wording of the items also differed slightly between studies. Although attempts are made to harmonize the single items by simple algorithmic transformation, it is unclear whether the items will in fact function equivalently across studies. Additionally, alcohol use norms also change with age, which requires allowing the parameters of the model to be moderated by study and age. To solve these issues, they first conducted a nonlinear factor analysis without moderation (i.e., assuming all parameters to be equal over groups). Then they extended this model by permitting the factor mean and variance to vary both as a function of (continuous) age and (discrete) study. Possible DIF were evaluated over study and age by simultaneously testing whether these two variables moderated the intercepts or factor loadings of the indicators. Finally, alcohol involvement scores were generated across all timepoints based on the estimates. |

[1]	Hong C, Liu Z, Liu Y, Jin Y, Luo Y. The Role of Smoking, Obesity, and Physical Inactivity in Cognitive Performance and Decline: A Multicohort Study. The Journals of Gerontology: Series A 2023;79:glad232. https://doi.org/10.1093/gerona/glad232.

[2]	Mendonça N, Hengeveld LM, Visser M, Presse N, Canhão H, Simonsick EM, et al. Low protein intake, physical activity, and physical function in European and North American community-dwelling older adults: a pooled analysis of four longitudinal aging cohorts. The American Journal of Clinical Nutrition 2021;114:29–41. https://doi.org/10.1093/ajcn/nqab051.

[3]	Ruiz M, Malyutina S, Pajak A, Kozela M, Kubinova R, Bobak M. Congruent relations between perceived neighbourhood social cohesion and depressive symptoms among older European adults: An East-West analysis. Social Science & Medicine 2019;237:112454. https://doi.org/10.1016/j.socscimed.2019.112454.

[4]	Callina KS, Johnson SK, Tirrell JM, Batanova M, Weiner MB, Lerner RM. Modeling Pathways of Character Development across the First Three Decades of Life: An Application of Integrative Data Analysis Techniques to Understanding the Development of Hopeful Future Expectations. J Youth Adolescence 2017;46:1216–37. https://doi.org/10.1007/s10964-017-0660-1.

[5]	Marcoulides KM, Grimm KJ. Data Integration Approaches to Longitudinal Growth Modeling. Educational and Psychological Measurement 2017;77:971–89. https://doi.org/10.1177/0013164416664117.

[6]	Curran PJ, Hussong AM, Cai L, Huang W, Chassin L, Sher KJ, et al. Pooling data from multiple longitudinal studies: The role of item response theory in integrative data analysis. Developmental Psychology 2008;44:365–80. https://doi.org/10.1037/0012-1649.44.2.365.

[7]	Bauer DJ, Hussong AM. Psychometric approaches for developing commensurate measures across independent studies: Traditional and new models. Psychological Methods 2009;14:101–25. https://doi.org/10.1037/a0015583.
